# Skeletal Muscle Loss During Treatment With Abiraterone in Patients With Metastatic Prostate Cancer

**DOI:** 10.1155/proc/1468262

**Published:** 2025-05-19

**Authors:** Eva Streckova, Jiri Stejskal, Daniela Kuruczova, Adam Svobodnik, Radka Stepanova, Tomas Buchler

**Affiliations:** ^1^Department of Oncology, Second Faculty of Medicine, Charles University, Prague, Czech Republic; ^2^Department of Oncology, Second Faculty of Medicine, Motol University Hospital, Prague, Czech Republic; ^3^Department of Oncology, First Faculty of Medicine, Charles University, Prague, Czech Republic; ^4^Department of Oncology, First Faculty of Medicine, Thomayer University Hospital, Prague, Czech Republic; ^5^Department of Urology, First Faculty of Medicine, Charles University, Prague, Czech Republic; ^6^Department of Urology, First Faculty of Medicine, Central Military Hospital, Prague, Czech Republic; ^7^ANOVA CRO, Prague, Czech Republic; ^8^Department of Pharmacology, Faculty of Medicine, Masaryk University, Brno, Czech Republic

**Keywords:** abiraterone, androgen deprivation therapy, prostate cancer, sarcopenia, skeletal muscle

## Abstract

**Background:** Abiraterone acetate is an androgen-receptor pathway inhibitor commonly used for treatment of metastatic prostate cancer. The levels of androgens during treatment with abiraterone acetate with prednisone (AAP) are lower than those achieved by androgen-deprivation therapy only, potentially resulting in a high risk of skeletal muscle loss.

**Methods:** The cohort included 43 patients treated with AAP for metastatic hormone-sensitive prostate cancer or metastatic castration-resistant prostate cancer. To detect and quantify sarcopenia, we utilized standard computer tomography (CT) imaging. Skeletal muscle mass index (SMI) was evaluated by assessing two adjacent axial sections at the level of the L3 vertebra.

**Results:** Sarcopenia at the time of AAP initiation was present in 72.1% of patients. Body mass index (BMI) was inversely associated with the presence of sarcopenia at the time of AAP initiation. There was a statistically significant decrease in SMI over AAP treatment. Age > 75 years and the absence of previous radiotherapy were associated with a higher rate of SMI decrease during AAP therapy. Overall and progression-free survival was not significantly associated with SMI decrease during AAP therapy.

**Conclusions:** SMI decline occurs during AAP treatment for mHSPC and mCRPC, and is more pronounced in patients over 75 years old and those without previous local treatment. There was no statistically significant association between survival outcomes and SMI decline during AAP therapy.

## 1. Introduction

Sarcopenia is defined as the loss of skeletal muscle mass (SM) accompanied by a decrease in muscle strength and physical performance [1]. Recent research suggests a notable interplay between body composition and therapeutic outcomes in many types of malignancies [2].

In addition to primary sarcopenia as a pathological manifestation of aging, secondary sarcopenia due to androgen deprivation is a concern in patients with metastatic prostate cancer. Androgen deprivation therapy (ADT) is a primary treatment modality for men with prostate cancer. One of the main extraprostatic side effects of ADT includes its impact on skeletal muscle health, with men aged 70 years and above exhibiting more pronounced decreases in SM compared to their younger counterparts [3, 4]. It has also been demonstrated that muscle quality significantly deteriorated in men undergoing ADT [5].

Abiraterone acetate inhibits 17α-hydroxylase/C17,20-lyase, resulting in decreased synthesis of androgens including testosterone and dihydrotestosterone [6]. The levels of androgens during treatment with abiraterone acetate with prednisone/prednisolone (AAP) are therefore even lower than those achieved by ADT only, creating the so-called “super-castrate” status [7]. In addition, prednisone is an obligatory part of abiraterone treatment, and corticosteroids are a known contributing agent to sarcopenia [8].

For these reasons, patients treated with abiraterone AAP may be at a particularly high risk of sarcopenia and its complications. The aim of the present study was to evaluate changes in muscle mass during therapy with AAP and ADT.

## 2. Patients and Methods

### 2.1. Patients

The study included 43 patients with metastatic prostate cancer who were on therapy with AAP between October 2014 and November 2021. The group consisted of 14 patients with metastatic hormone-sensitive prostate cancer (mHSPC) and 29 patients with metastatic castration-resistant prostate cancer (mCRPC). The analysis was carried out retrospectively. All patients signed informed consent with the therapy as well as data analysis. The inclusion criteria for the study were treatment with AAP for metastatic prostate cancer, the availability of computed tomography (CT) scans acquired at the time of initiation of AAP and another CT scan at a later timepoint, and evaluable data on baseline parameters and survival. CT scans were required to be evaluable for the presence of sarcopenia at the level of third lumbar vertebra (L3) using a previously described procedure and quality criteria [9].

The study activities were conducted in accordance with the Declaration of Helsinki. All patients enrolled in the study signed informed consent with AAP therapy and with evaluation of their data for research purposes within a noninterventional study as approved by the Ethics Committee of the Thomayer University Hospital. Subject data were analyzed retrospectively and personal information were pseudonymised in accordance with EU general data protection regulation 2016/679 (GDPR).

### 2.2. Muscle Mass Measurement

To detect and quantify sarcopenia, we utilized standard computer tomography (CT) imaging methods. The diagnostic abdominal CT scans were used for the clinical assessment of disease progression, thus not imposing additional ionizing radiation burden on the patients for sarcopenia measurement purposes. Sarcopenia quantification was performed using software analysis with the National Institutes of Health (NIH) ImageJ program. Following the recommendations for the analysis of diagnostic CT sections using NIH ImageJ, we evaluated two adjacent axial sections at the level of the L3 vertebra. Muscle contouring at this level was done manually. From the sections, we calculated the total cross-sectional area of the muscles at this level (i.e., m. rectus abdominis, m. transversus abdominis, m. obliquus abdominis internus et externus, m. psoas, m. quadratus lumborum, m. erector spinae) using density settings for muscle tissue. The measured muscle area was then normalized to the patient's height, resulting in the lumbar skeletal muscle index (SMI). Sarcopenia was defined as an SMI at *L*3 ≤ 52.4 cm^2^/m^2^ [10]. Because of the arbitrary time between the two CT scans, time elapsed between CT scans was considered in all models.

### 2.3. Statistical Analysis

For continuous variables, the mean, standard deviation, median, and range were reported. Categorical variables were described using absolute and relative frequencies. To determine the impact of various clinical variables on the evolution of SM, a linear mixed effects model was employed. The model used measured muscle mass as the dependent variable, with time and any additional variables as fixed effects. Patient identification number was set as the random effect with unstructured covariance. To identify variables that are associated with muscle mass decrease over time, a separate mixed effects model was constructed for each variable. Subsequent analyses included only variables where at least a weak association with the change in muscle mass could be demonstrated. The selection of variables for the final model also included analysis of association between candidate variables to avoid multicollinearity. AAP treatment duration was used as a surrogate for progression-free survival (PFS), as AAP therapy is not reimbursed after progression. For the analysis of overall survival (OS) and PFS, a dichotomous variable was created for each patient—loss of muscle mass below and above 1% per month of treatment. To compare patients within these subgroups the Kaplan–Meier estimator was used, with log-rank test for subgroup comparison.

## 3. Results

### 3.1. Baseline Characteristics

In the cohort of 43 patients enrolled in the study, the mean initial PSA was 341.08 ng/mL (range. 1.60 to > 5000 ng/mL). The treatment indication was mHSPC in 14 patients and mCRPC in 29 patients. Standard androgen deprivation was maintained in all patients, with testosterone levels < 50 ng/mL. The dose of abiraterone acetate was 1000 mg daily orally with prednisone 10 mg daily orally. The mean time between CT scans was 14.33 months (3.01–63.17 months).

The stage at prostate cancer diagnosis was localised disease (T1-2 N0 M0) in four patients (9.30%), locally advanced disease (T3-4 or N1, M0) in 10 patients (23.25%), and metastatic disease in 29 patients (67.44%). Luteinizing hormone-releasing hormone (LHRH) receptor agonists were used as ADT in 27 patients (62.80%) and bilateral orchiectomy in 16 patients (37.20%). By the data cutoff date, 31 patients (72.09%) have discontinued AAP treatment with the median duration of AAP therapy of 8.28 months (range 3.09–82.33 months).

Other baseline characteristics of the cohort are shown in Table 1.

### 3.2. Changes of SM During Treatment With Abiraterone

Sarcopenia at the time of AAP initiation was present in 72.1% of patients. Body mass index (BMI) was inversely associated with the presence of sarcopenia at the time of AAP initiation. There was no statistically significant difference between the groups of patients with mHSPC and mCRPC. The decrease in SMI was not significantly influenced by the patient's initial weight, BMI, previous chemotherapy, or sarcopenia present at treatment initiation (Table 2 and Supporting Table 1).

There was an overall statistically significant decrease of SMI during AAP treatment. Age over 75 was associated with more rapid SM reduction. Previous radiotherapy was associated with lower risk of skeletal muscle loss during AAP treatment. These results are summarised in Table 2, showing the final mixed effect model based on multivariable analysis.

### 3.3. Survival Analysis

**Figure 1:**
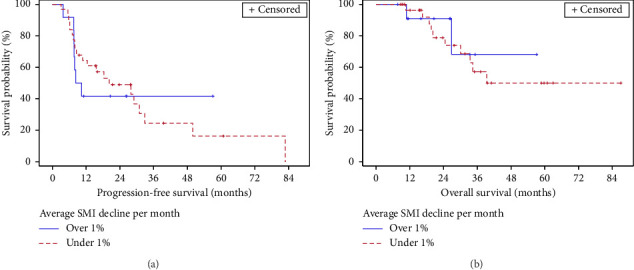
Progression-free survival (a) and overall survival (b) by skeletal muscle loss during treatment (average monthly skeletal muscle loss of > 1% vs. others).

OS and PFS were compared for subgroups with an average decline in SMI per month of treatment above 1% versus others (Figure 1). The difference between these two groups was not significant for either outcome according to the log-rank test, despite numerical differences in PFS indicating a trend to worse outcome in patients with more rapid SMI decrease (Table 3).

## 4. Discussion

The presence of sarcopenia is common in men undergoing therapy with AAP. A significant proportion of these patients are sarcopenic at the onset of the therapy and there is a significant loss of muscle mass during the treatment. According to the present analysis, the decline was more pronounced in patients over 75 years old. Higher BMI was associated with lower risk of sarcopenia at treatment initiation. However, the reduction of SM during AAP therapy was not associated with initial BMA, initial sarcopenia, or HSPC/CRPC status. Previous surgery or radiotherapy was associated with lower risk of sarcopenia. According to our analysis, there was no statistically association between PFS or OS and the SMI decrease during AAP therapy, although there was a numerical difference indicating lower PFS in patients with more pronounced average monthly SMI decrease.

Collectively, published studies highlight the potential of body composition parameters, particularly sarcopenia, as critical prognostic markers in advanced prostate cancer, warranting further interventional studies and standardization of definitions to inform clinical decision-making [11–15]. The association has been established for many systemic treatment modalities used for the therapy of metastatic prostate cancer, including radioligand therapy [16, 17] and chemotherapy [18–21].

Chiang et al. linked muscle loss during ADT with increased noncancer mortality in high-risk prostate cancer patients [22]. A systematic review by de Pablos-Rodríguez et al. published in 2022 reinforced the prognostic value of sarcopenia in advanced prostate carcinoma, finding a significant correlation between sarcopenia and shorter PFS [23].

However, not all studies confirm the negative association of sarcopenia and survival in prostate cancer. Mason et al. showed no significant association between sarcopenia and oncologic outcomes post-radical prostatectomy, suggesting the condition may not influence outcomes for localized prostate cancer treatments [24]. Hiroshige and collaborators found that among mCRPC patients treated with androgen receptor axis-targeted therapies, those with initial sarcopenia showed better PFS rates than their nonsarcopenic counterparts, marking sarcopenia as a potentially favorable prognostic factor in this context [25]. In a phase II clinical trial with neoadjuvant chemohormonal therapy, despite changes in body composition including the development or worsening of sarcopenia, no significant correlation was found between these alterations and disease progression [21]. Similarly, skeletal muscle loss during therapy was not associated with PFS or OS in our study. These results underscore the multifaceted interplay between prostate cancer treatments, body composition, and patient outcomes, revealing a pressing need for personalized patient management strategies. The etiology of sarcopenia in this population is multifactorial, with the most important determinants being older age, sedentary lifestyle, poor nutritional status, cardiovascular comorbidities, and especially metastatic prostate cancer itself, a disease associated with poor bone health due to metastases and treatment-related osteopenia. Larger future studies could address differences in these important confounding factors through patient stratification.

Due to its powerful inhibitory effect on residual androgen synthesis in the setting of ADT, androgen-receptor pathway inhibitors appear to accelerate the process of skeletal muscle loss. Fischer and collaborators found that AAP and enzalutamide used in the setting of mCRPC were associated with a significant reduction in skeletal muscle. Interestingly, while both drugs induced a comparable muscle loss, they did not significantly alter subcutaneous fat levels [26].

A post hoc analysis was conducted on patients treated with AAP in clinical trials by Pezaro and collaborators who showed that treatment with AAP led to muscle loss, with the highest loss in patients having a baseline BMI over 30. Visceral fat loss was also observed with AAP, most prominently in patients with a baseline BMI over 30. Adding dexamethasone resulted in a marked increase in central fat in patients, regardless of their baseline BMI. Despite the addition of dexamethasone, there was no further significant loss of SM [27].

A recently published study examined the effects of different ADT regimens on body composition in prostate cancer patients, highlighting the distinct metabolic impacts of different forms of endocrine treatment. It focused on changes in muscle and fat tissues over 18 months among 229 patients treated with standard LHRH agonists (aLHRHs) alone or combined with newer agents like AAP or enzalutamide. Results indicated that AAP and enzalutamide led to more significant muscle loss than aLHRHs, with AAP also increasing visceral adipose tissue [28]. Early detection of sarcopenia and the integration of tailored interventions, such as resistance-based exercise programs, could significantly benefit prostate cancer patients undergoing endocrine treatments [29].

Major limitations for the present study include small size of the patient cohort, lack of a control group, and retrospective design. The cutoff value has not been defined and validated for patients with metastatic prostate cancer in the Czech population. We used the cutoff value developed for cancer patients by Prado et al. and accepted by many authors [10, 30–32]. Although detailed description of the cutoff values used for sarcopenia in previously published studies is outside the scope of the present paper, detailed overviews have been published [33, 34].

To narrow the knowledge gaps regarding sarcopenia in patients with metastatic prostate cancer, future research should focus on several specific areas to deepen the understanding of the relationships and mechanisms involved. Longitudinal studies with larger cohorts could determine if certain demographic or clinical characteristics (such as age, baseline sarcopenia status, or prior therapies) influence the risk of developing sarcopenia during treatment. This can help identify high-risk groups who might benefit from early intervention. They could also better define inflammatory cytokines, hormonal changes, and metabolic disruptions that may mediate sarcopenia in these patients and find biomarkers for skeletal muscle loss.

Controlled trials could directly compare the effects of different therapies on muscle mass and function. They could include comparisons between AAP and other therapies such as enzalutamide or newer androgen receptor inhibitors to ascertain differential impacts on muscle health. Such studies could identify the optimal treatment agent for prostate cancer patients with pre-existing sarcopenia.

There is also a need for prospective studies testing intervention strategies such as resistance training, nutritional support, and pharmacological agents aimed at mitigating muscle loss. Finally, studies should evaluate the economic impact of sarcopenia in prostate cancer patients, including healthcare costs associated with increased morbidity, treatment modifications, and the potential economic benefits of effective sarcopenia management strategies.

In conclusion, the present study highlights the high incidence of sarcopenia in patients undergoing treatment with AAP and further decrease of skeletal mass during the treatment. The findings highlight the importance of monitoring and addressing body composition changes in men with advanced prostate cancer, especially when considering the potential ramifications on the quality of life and other health risks. Given the relatively small number of evaluated patients, a more extensive analysis is desirable. For high-risk patients, initiation of sarcopenia prevention and treatment with appropriate nutritional intervention and physical activity should be considered.

## Figures and Tables

**Table 1:** Baseline patients' characteristics.

Parameter	*N* = 43
Height (cm)	
Mean (SD)	177.2 (7.35)
Median	176
Range	158–194
Sarcopenic at onset	
No	12 (27.9%)
Yes	31 (72.1%)
Age (years)	
Mean (SD)	69.1 (8.11)
Median	70
Range	47–83
Age over 75 years	
No	32 (74.4%)
Yes	11 (25.6%)
Initial weight (kg)	
Mean (SD)	87.8 (14.77)
Median	86
Range	59–130
Weight over 100 kg	
No	33 (76.7%)
Yes	10 (23.3%)
Initial BMI (kg/m^2^)	
Mean (SD)	27.94 (4.22)
Median	27.12
Range	20.22–40.12
BMI over 30 (kg/m^2^)	
No	32 (74.4%)
Yes	11 (25.6%)
Sarcopenia and obesity at onset	
No	38 (88.4%)
Yes	5 (11.6%)
ADT type	
LHRH	26 (60.5%)
Orchiectomy	17 (39.5%)
CRPC (1)/HSPC (2)	
1	30 (69.8%)
2	13 (30.2%)
Meta bones	
No	5 (11.6%)
Yes	38 (88.4%)
Meta visceral	
No	38 (88.4%)
Yes	5 (11.6%)
Previous prostatectomy	
No	39 (90.7%)
Yes	4 (9.3%)
Previous radiotherapy	
No	33 (76.7%)
Yes	10 (23.3%)
Previous chemotherapy	
No	30 (69.8%)
Yes	13 (30.2%)
Initial ISUP grade	
2	2 (4.9%)
3	6 (14.6%)
4	13 (31.7%)
5	20 (48.8%)
Missing	2 (%)

Abbreviations: ADT, androgen deprivation therapy; BMI, body mass index; CRPC, castration-refractory prostate cancer; HSPC, hormone-sensitive prostate cancer; ISUP, International Society of Urological Pathology; SD, standard deviation.

**Table 2:** Final mixed effects model for the presence of sarcopenia at the onset of AAP therapy and for the decrease of SMI during AAP therapy. Higher initial BMI was associated with lower probability of sarcopenia at the onset of treatment. Absence of previous radiotherapy and age > 75 years were associated with SMI decrease during the treatment.

Effect	Category	Estimate (SD)	*p* value
Initial BMI		0.819 (0.244)	0.0017
SMI decrease over time		−0.010 (0.001)	< 0.0001
SMI decrease over time (age > 75 years)	Yes	−0.009 (0.004)	0.0218
SMI decrease over time (previous radiotherapy)	Yes	0.008 (0.002)	< 0.0001

Abbreviations: AAP, abiraterone acetate and prednisone; BMI, body mass index; SMI, skeletal muscle index.

**Table 3:** Overall survival and progression-free survival by average monthly SMI decrease during AAP therapy.

Parameter	SMI loss > 1% per month (*N* = 12)	Others (*N* = 31)
PFS events (*N*)	7 (58.3%)	21 (67.7%)
OS events (*N*)	2 (16.7%)	10 (32.3%)
PFS (months)		
Mean (SD)	16.25 (14.95)	21.11 (17.87)
Median	9.37	15.64
Min-max	3.80–57.12	3.11–82.84
OS (months)		
Mean (SD)	23.57 (14.03)	30.02 (19.23)
Median	23.40	24.53
Min-max	7.74–57.12	8.63–87.04

Abbreviations: OS, overall survival; PFS, progression-free survival; SD, standard deviation; SMI, skeletal muscle index.

## Data Availability

Data will be provided by the authors upon reasonable request.

## Supporting Information

Supporting Table 1. Univariate analysis for skeletal muscle mass loss during therapy with abiraterone acetate and prednisone.
