# Supplementary material for: Skeletal Muscle Loss During Treatment With Abiraterone in Patients With Metastatic Prostate Cancer
Source: Prostate Cancer. 2025 May 19;2025:1468262. doi: 10.1155/proc/1468262 (PMC12105893; doi:10.1155/proc/1468262)
Supplement: Supporting Information — Additional supporting information can be found online in the Supporting Information section. [file 1468262.f1.docx]

**Supplementary Table**

Supplementary Table 1. **Univariate analysis for skeletal muscle mass loss during therapy with AAP**

| Effect | Category | Estimate (SD) | P-value |
| --- | --- | --- | --- |
| Sarcopenic at onset | Yes | 0.001 (0.002) | 0.7988 |
| **Age over 75** | **Yes** | **-0.011 (0.005)** | **0.0248** |
| **Weight over 100** | **Yes** | **0.005 (0.002)** | **0.0289** |
| BMI over 30 | Yes | 0.003 (0.002) | 0.1541 |
| Sarcopenia and Obesity at onset | Yes | -0.003 (0.004) | 0.4858 |
| **ADT type** | **LHRH** | **-0.005 (0.002)** | **0.0091** |
| CRPC (1) / HSPC (2) | 1 | 0.005 (0.002) | 0.0584 |
| Meta bones | Yes | -0.002 (0.005) | 0.7498 |
| Meta visceral | Yes | -0.002 (0.007) | 0.7059 |
| **Previous prostatectomy** | **Yes** | **0.006 (0.002)** | **0.0163** |
| **Previous radiotherapy** | **Yes** | **0.008 (0.002)** | **<.0001** |
| Previous chemotherapy | Yes | 0.003 (0.002) | 0.2466 |
| Initial ISUP grade | 2 | -0.021 (0.017) | 0.2311 |
|  | 3 | 0.004 (0.003) | 0.1677 |
|  | 4 | 0.005 (0.003) | 0.0692 |
| Height (cm) |  | 0 (0) | 0.6583 |
| Age (years) |  | 0 (0) | 0.2381 |
| Initial weight |  | 0 (0) | 0.1921 |
| Initial BMI (kg) |  | 0 (0) | 0.2163 |
